# Cooperation of GlycoPOST and UniCarb-DR towards a comprehensive glycomics data repository workflow

**DOI:** 10.1007/s00216-024-05673-3

**Published:** 2024-11-29

**Authors:** Yushi Takahashi, Niclas G. Karlsson, Shujiro Okuda, Kiyoko F. Aoki-Kinoshita

**Affiliations:** 1 Medical AI Center, Niigata University School of Medicine, Niigata, Japan; 2 Division of Pharmacy, Department of Life Sciences and Health, Faculty of Health Sciences, Oslo Metropolitan University, Oslo, Norway; 3 Glycan and Life Systems Integration Center, Faculty of Science and Engineering, Soka University, Tokyo, Japan

**Keywords:** Glycomics, Glycoinformatics, Mass spectrometry, Liquid chromatography, Metadata, Data repository

## Abstract

In glycomics, two data repositories, GlycoPOST and UniCarb-DR, have been developed to accumulate experimental data generated by glycomics and glycoproteomics mass spectrometry experiments. In order to enhance the interrelation between these two data repositories, we have upgraded the framework for both of them; we have unified their respective data submission systems and constructed a mechanism that can automatically cross-reference corresponding entries. In addition to this integration, the metadata registration system was also extended so that liquid chromatography experiments can be reported according to standard reporting guidelines specified by MIRAGE (Minimum Information Required for A Glycomics Experiment). Furthermore, by augmenting the visualization software used in UniCarb-DR, we have been able to introduce new functionality into GlycoPOST to enable the visualization of unpublished experimental identification result files during an embargo period defined by the data provider. As a result, this work introduces a new framework by which glycomics researchers can take advantage of GlycoPOST and UniCarb-DR in an integrated manner.

**Graphical abstract:**

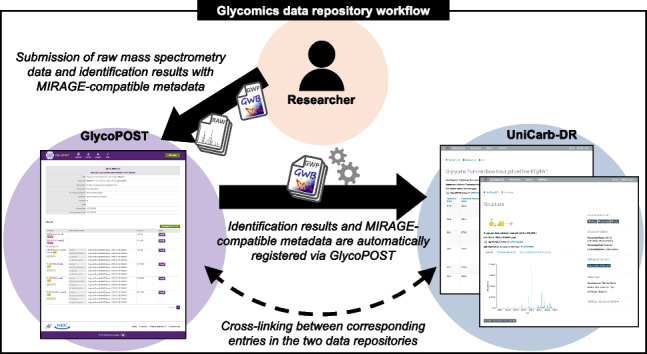

**Supplementary Information:**

The online version contains supplementary material available at 10.1007/s00216-024-05673-3.

## Introduction

Glycosylation is a biochemical reaction in which monosaccharides or oligosaccharides are attached to host proteins and is one of the major examples of post-translational modifications on proteins. Among various post-translational modifications, glycosylation is attracting more attention because it is known to be closely related to various biological mechanisms such as folding of host proteins, intercellular communication, virus infection, development, and diseases such as cancer and muscular dystrophy [1–6]. Protein glycosylation is mainly divided into two classes: *N*-linked glycans attached to the nitrogen atom of an asparagine residue in the protein, and *O*-linked glycans attached to the oxygen atom of a serine or threonine residue [7]. Glycosylated proteins are presented on the cell surface or secreted outside the cell and are known to interact with glycan-binding proteins (GBPs) to mediate various reactions such as cell-cell interactions and extracellular matrix interactions [2]. Recently, advances in glycoproteomics have implied that *N*-glycosite mapping can be used for disease diagnosis [8, 9]. Furthermore, *N*-glycoproteomic analysis has revealed that neurological diseases such as Alzheimer's disease have different *N*-glycosylation patterns than healthy controls [8, 10].

As a tool for identifying the structures of glycans that appear on proteins or peptides, mass spectrometry is an essential analytical tool in glycomics, allowing highly sensitive detection of small amounts of samples. Glycans released from core proteins using glycosidases (such as PNGase F), or chemically (e.g., β-elimination), can be identified by liquid chromatography-tandem mass spectrometry (LC-MS/MS). Today, in the life sciences, it is becoming increasingly important to accumulate various experimental data, including raw mass spectrometry data, in public data repositories and share them to improve the reproducibility of experiments and to facilitate comprehensive reanalysis of experimental results.

For various omics fields, there are several public data repositories where researchers can submit their experimental data according to the FAIR (Findable, Accessible, Interoperable, and Re-usable) data principles [11]. GenBank [12] in genomics, and Proteomics Identification Database (PRIDE) [13], PeptideAtlas [14], and jPOST repository [15], members of the ProteomeXchange consortium [16], in proteomics are widely known. In glycomics, two data repositories have been developed, GlycoPOST [17] (https://glycopost.glycosmos.org/) and UniCarb-DR [18] (https://unicarb-dr.glycosmos.org/), both of which are currently hosted on the GlyCosmos Portal [19]. GlycoPOST accepts raw data files generated from mass spectrometers used in glycomics experiments, along with peak list files and identification results, and, as of August 25, 2024, 357 projects have been registered, with a total of 20,864 files and a total data size of 3.5 terabytes. On the other hand, UniCarb-DR can accept mass spectrometry identification results annotated with GlycoWorkbench [20], which is software used for identifying glycans from mass spectrometry experiment files in glycomics, allowing researchers to visualize their results graphically on a web browser and to search for identification results based on various criteria.

Because biological mass spectrometry results depend on sample preparation methods and types of instruments used, experimental results from mass spectrometers obtained under different conditions cannot be directly compared with one other. Therefore, in order to confirm the reproducibility of the large amount of mass spectrometry data deposited in data repositories and to properly facilitate knowledge discovery through exhaustive computer-based reanalysis, a wide range of metadata assigned to the data is required. In glycomics, there is a set of standardized guidelines for reporting experimental results called MIRAGE (Minimum Information Required for A Glycomics Experiment) [21], and, at the time of this writing, eight guidelines have been proposed, including guidelines for sample preparation [22], liquid chromatography analysis [23], and mass spectrometry analysis [24]. Both GlycoPOST and UniCarb-DR have been developed with the MIRAGE sample preparation and mass spectrometry guidelines in mind, allowing researchers to annotate and submit experimental data files with reporting guidelines metadata.

Historically, the GlycoPOST and UniCarb-DR repositories have been separate and independent systems developed and maintained by different institutions. This led to a dual submission process, uploading data to each repository separately and obtaining independently assigned accession numbers. Therefore, even if the entries were for the same experimental result, it was difficult to associate the corresponding entries with each other. For example, if a user wanted to find a raw data file for an identification result registered in UniCarb-DR, it would be quite time-consuming to find out whether the data is registered in GlycoPOST or not, and if so, to obtain its accession number. In addition, while GlycoPOST allows researchers to freely set the publication date of their submitted data, allowing only reviewers to access them during the peer review period (i.e., the embargo period) of their papers, the embargo was not possible in previous versions of UniCarb-DR. The first step in communication between the two repositories was established in 2018, when a common login system was developed and implemented at GlyCosmos. In this study, we went further and enhanced the interoperability of these two data repositories and unified their data submission systems. The ambition is to make it easier for glycomics researchers to register and utilize the experimental results. As a result, researchers can now leverage both of the data repositories to narrow down candidates for raw mass spectrometry data that have similar identification results. In addition, in order to support the registration of metadata regarding liquid chromatography experiments, previously only partially supported by UniCarb-DR, we have extended both the GlycoPOST and UniCarb-DR systems to allow this type of experiment metadata to be entered according to the latest liquid chromatography guidelines proposed by MIRAGE [23].

UniCarb-DR can visualize the mass spectrometry identification results required by the MIRAGE MS guidelines in a web browser in an intuitive manner, while GlycoPOST is focused on hosting submitted experimental data, and therefore has little functionality for visualizing them. With the new data submission flow established in this study, identification result files using the GlycoWorkbench format included in projects published on GlycoPOST are now automatically registered and visualized in UniCarb-DR. Furthermore, we have also instituted so that both submitter and reviewers can have access and visualize the data during the embargo period if the MIRAGE required identification results were included in the files submitted to GlycoPOST. If a glycan structure included in the identification results is registered in GlyTouCan [25], the international glycan structure repository, it is now possible to automatically link from these two data repositories to the corresponding entry page in GlyTouCan.

## Materials and methods

### Data submission system integration of the two data repositories and enhanced integration with GlyTouCan

GlycoPOST, from the beginning, implemented a file submission system designed for fast uploading of all types of files, as well as application programming interfaces (APIs) that allowed external servers to retrieve metadata associated with files in any publicly available dataset (called “projects”). This file submission system was adopted to develop a unified data submission system shared between GlycoPOST and UniCarb-DR. As a means of sharing the data accumulated in GlycoPOST with UniCarb-DR, we established a system using the Scala programming language that automatically downloads the experiment identification result files in GlycoWorkbench format (hereinafter GlycoWorkbench files) in projects newly published on GlycoPOST and registers them in UniCarb-DR. This program was configured to run as a daily batch process by leveraging the continuous integration pipeline of GitLab running on an on-premise server.

We have also interoperability with GlyTouCan so that each identification result registered in UniCarb-DR can be displayed on the entry screen of UniCarb-DR with its GlyTouCan ID if the corresponding glycan structure has been registered in GlyTouCan. To implement this functionality, we used an API originally developed at the Swiss Institute of Bioinformatics (SIB) and incorporated it into the GlyCosmos Portal to search for GlyTouCan IDs from glycan structure text in linear format used in GlycoWorkbench (.gws) [18]. This API is used to obtain GlyTouCan IDs for any glycans identified in GlycoWorkbench, which is then used in these two repositories.

### Support of MIRAGE liquid chromatography guidelines

In order to add support for the liquid chromatography guidelines proposed by MIRAGE, to GlycoPOST and UniCarb-DR, we have updated GlycoPOST's user interface written mainly in JavaScript language and API written in the PHP language. At the same time, we registered additional data into the GlycoPOST MySQL database so that new metadata information based on the guidelines could be entered. Furthermore, a server-side program for UniCarb-DR was written in the Scala language to enable registration of this new metadata to UniCarb-DR via GlycoPOST's API. Accordingly, the user interface of UniCarb-DR was also updated to allow browsing of the new metadata introduced from GlycoPOST.

### Identification results visualization during the embargo period

We extracted the glycan structure rendering and spectral viewer functionality of UniCarb-DR, which is written in the Scala and JavaScript languages, as a reusable component, naming it MiniCarb-Viewer, and we introduced it into the GlycoPOST project preview screen in a private data rendering manner. This tool allows users to visualize the glycans in GlycoWorkbench files included in projects during the embargo period on GlycoPOST similar to UniCarb-DR. Since the size of GlycoWorkbench files varies greatly for each file (from tens of kilobytes to hundreds of megabytes), a synchronous registration process could potentially delay server response time. To avoid such issues, we developed a server-side program to register GlycoWorkbench files asynchronously in the background by adopting an asynchronous processing library called Apache Pekko.

## Results and discussion

### New unified submission workflow

**Fig. 1:**
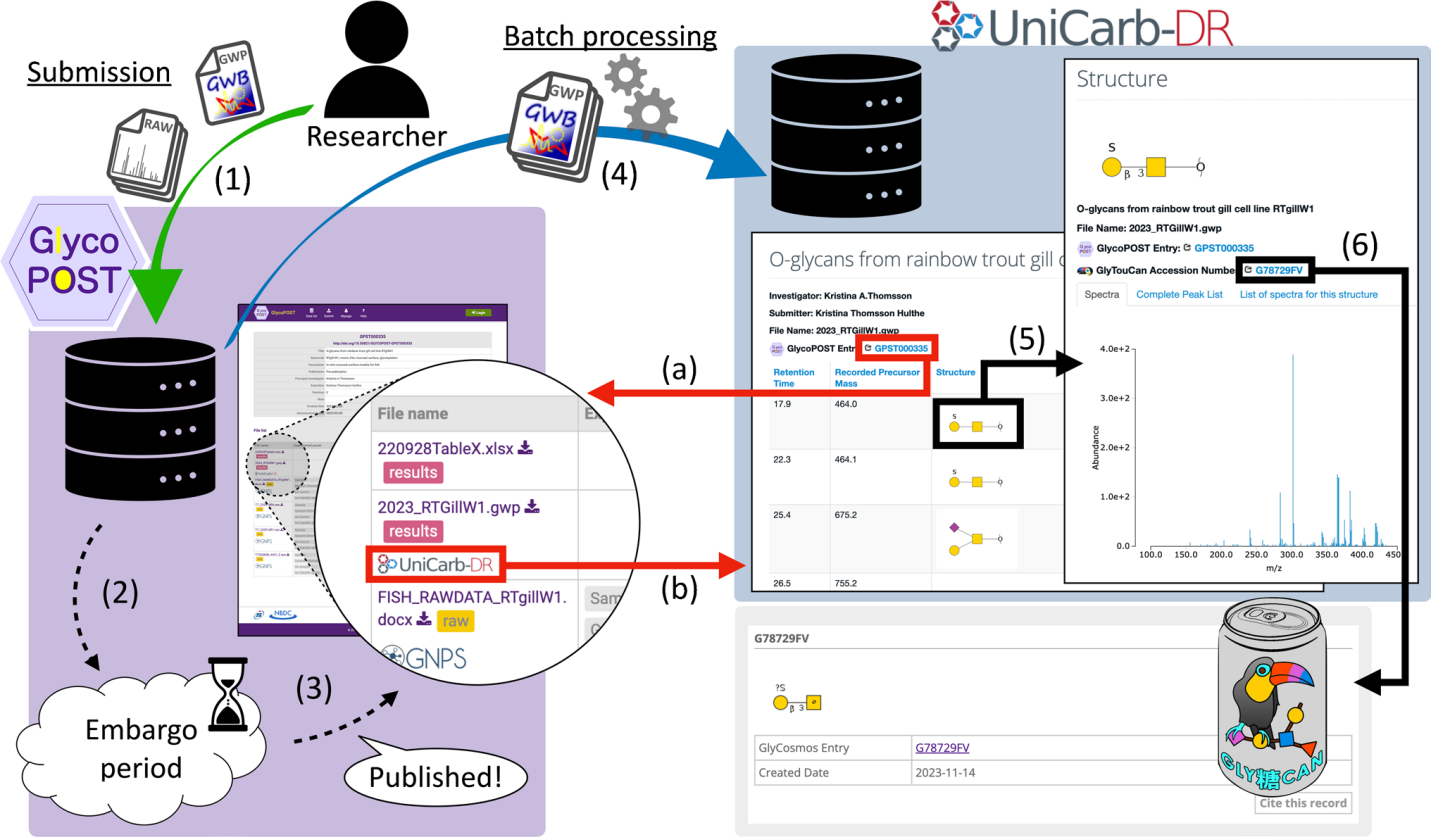
Conceptual diagram of the unified data submission flow to the two data repositories. The green arrow indicates the submission of experimental data including GlycoWorkbench files (GWP files) from researchers, and the blue arrow indicates the download of GlycoWorkbench files via our batch process. The black dashed lines indicate the passage of time during the embargo period, and the black and red solid lines indicate hyperlinks between web pages. The details of each step, represented by the parenthesized numbers in this diagram, are described in the text of the “Results and discussion” section. In particular, the solid red lines indicated as (a) and (b) refer to the cross-reference of corresponding entries between GlycoPOST and UniCarb-DR. In addition, if a GlyTouCan ID corresponding to a glycan structure registered in UniCarb-DR exists, a link to the entry corresponding to that ID is also displayed

Figure 1 shows a conceptual diagram of the unified data submission flow between the two data repositories newly established in this work and the collaboration between these repositories and GlyTouCan. Previously, researchers had to submit their mass spectrometry results separately to GlycoPOST and UniCarb-DR. In this new submission flow, they can upload all data, including GlycoWorkbench files, together in GlycoPOST instead of UniCarb-DR (Fig. 1 (1)). During the peer review process (i.e., the embargo period), these submitted data are accessible only by submitters and reviewers, and are not available to the public (Fig. 1 (2)). Researchers can freely set and modify their data publication date via their web browsers, but only if the data is within the embargo period and not released to the public. After the embargo period is over and the experimental data are made publicly available on GlycoPOST (Fig. 1 (3)), the data containing GlycoWorkbench files that UniCarb-DR can interpret are automatically downloaded by the UniCarb-DR batch processing system and registered in its database in the background (Fig. 1 (4)). Once the registration process is complete, any users can easily review the contents of each GlycoWorkbench file registered in UniCarb-DR and browse detailed information, including spectral data, by clicking on a glycan structure image (Fig. 1 (5)), which is rendered using the Symbol Nomenclature for Glycans (SNFG) monosaccharide symbols [26]. If the glycan structure has already been registered in GlyTouCan, the corresponding GlyTouCan ID is displayed on the screen. Users can click on the ID to jump to the corresponding glycan structure entry page of GlyTouCan and browse its detailed information (Fig. 1 (6)).

With this mechanism, researchers can simply submit all the data related to an experiment into GlycoPOST, which will then automatically be published with the identification results of that experiment on UniCarb-DR after the paper is made publicly available, without the need to access UniCarb-DR. Since the size of GlycoWorkbench files varies widely from file to file, registration of large files in UniCarb-DR previously could fail due to timeouts on the web server. The new registration system automatically eliminates such problems. Moreover, we updated both systems to automatically cross-reference between GlycoPOST and the corresponding entries in UniCarb-DR (Fig. 1(a) and (b)), making it possible to visualize the identification results in GlycoPOST on UniCarb-DR. Conversely, it is also now possible to confirm from which raw data the identification results registered on UniCarb-DR were originally obtained by referring to GlycoPOST. Electronic Supplementary Material Table S1 lists the GlycoWorkbench files on UniCarb-DR that have been retrieved from GlycoPOST and registered according to the new data submission flow. As of August 25, 2024, GlycoPOST contains a total of 36 GlycoWorkbench files in public projects. Thirty-four of the 36 files were successfully registered in UniCarb-DR, and the remaining 2 files failed to read valid data for UniCarb-DR, resulting in errors.

As a result of the work described above, it is now possible to link from UniCarb-DR to the corresponding entry in GlyTouCan, though this is still only one-way linking. We plan to promote the collaboration among these three data repositories to enable bidirectional cross-referencing in the future. In addition, even if a GlycoWorkbench file contains a glycan structure that does not yet have a GlyTouCan ID, this workflow does not automatically register that structure into GlyTouCan. Therefore, we will soon implement a system that automatically registers such glycan structures found in GlycoWorkbench files registered in GlycoPOST into GlyTouCan and include it into our workflow.

### Novel search association of glycomics data enabled by the collaboration of the two data repositories

**Fig. 2:**
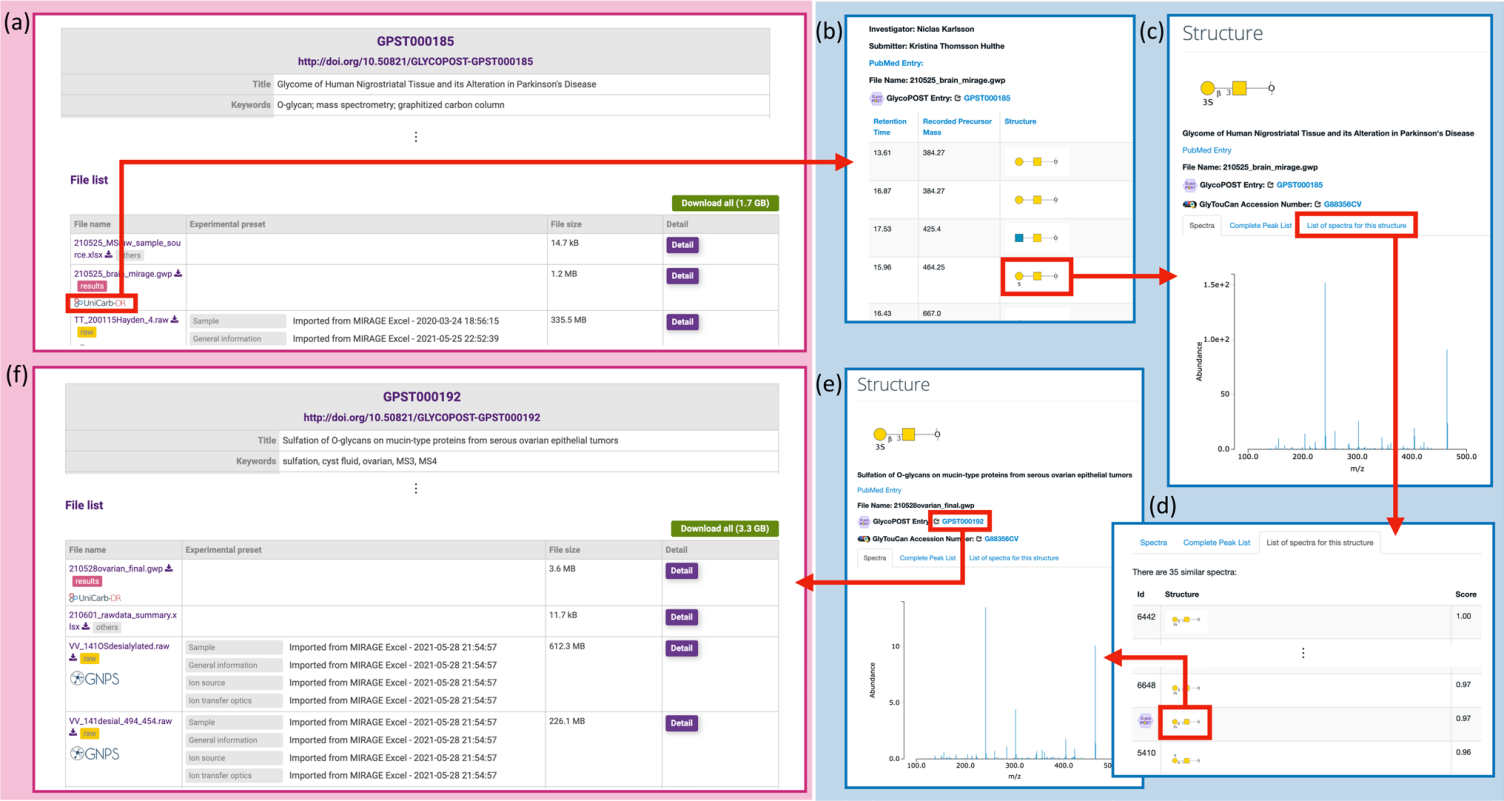
Procedure for searching raw data files that have generated similar spectral glycan structure identification results across the two data repositories. The images with purple background color on the left-hand side are screenshots of GlycoPOST, while the images with blue background color on the right-hand side represent screenshots of UniCarb-DR. **a** If a project published on GlycoPOST includes the identification results along with the raw data as a GlycoWorkbench file, a link to UniCarb-DR is displayed in the corresponding row in the table. **b** A series of experimental identification results contained in the GlycoWorkbench file are listed on UniCarb-DR. **c** When a glycan structure image representing a specific identification result is clicked in the list, the detail screen of the result is displayed. **d** By clicking on the “List of spectra for this structure” tab in that screen, other identification results that have a very similar spectrum to that result will be sorted and displayed in order of similarity score. **e** When an image of the glycan structure corresponding to a specific identification result is clicked from the list, the screen jumps to the detail screen of that clicked structure. If the data is derived from a GlycoWorkbench file obtained from GlycoPOST, the corresponding GlycoPOST project ID will be displayed on the screen. **f** Clicking on that project ID opens the corresponding project detail screen in GlycoPOST, which lists the raw data files for that experiment. In this fashion, researchers can traverse the two data repositories to find raw data files obtained from different experiments, but with very similar identification results

The enhanced collaboration between these data repositories enables glycoinformaticians to search for data across the two data repositories while taking full advantage of their respective features. For example, UniCarb-DR allows users to search for identification results from registered experiments that have spectra similar to a spectrum of interest. With this functionality, raw mass spectrometry data with similar identification results on GlycoPOST can be retrieved. Figure 2 shows the detailed workflow of this use case.

However, since a GlycoWorkbench file can contain identification results from multiple raw mass spectrometry data files, this search functionality needs additional improvement. Mitigating this limitation requires GlycoPOST to support additional metadata that describes the relationships between files, similar to the MAGE-TAB format for proteomics (MAGE-TAB-Proteomics) [27]. We believe that implementing such a relationship is a first step in establishing a methodology to comprehensively extract knowledge from data through informatics-based approaches such as machine learning. We are planning on developing a methodology for associating raw data, including metadata attached to each experimental data.

### Implementation of a new preset category for liquid chromatography experiment metadata into GlycoPOST

**Fig. 3:**
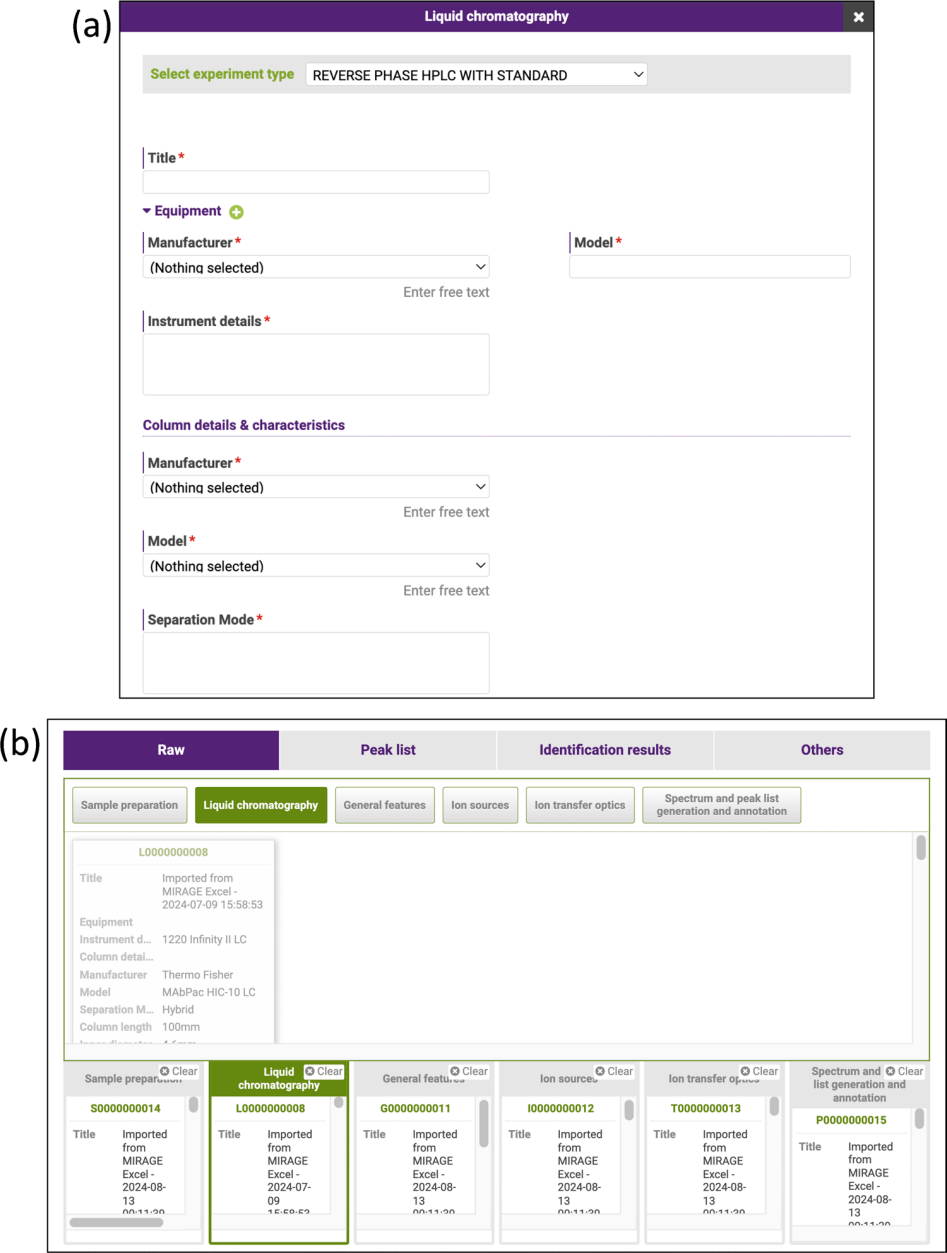
Screenshots showing the updated GlycoPOST screens for entering liquid chromatography–related metadata. **a** A screenshot of a part of the newly added web form for entering presets describing liquid chromatography experiments in GlycoPOST. Users can enter metadata related to liquid chromatography experiments, including experimental equipment, columns used, and mobile phases, on this form, following the MIRAGE liquid chromatography guidelines. **b** A screenshot of the GlycoPOST data upload screen, which now allows presets for liquid chromatography experiments to be associated with experimental data. Users can choose from each preset category at the top of the figure to associate the appropriate metadata, including liquid chromatography experiment information, with each experimental results file included in their submission. The bottom of this figure shows a preview of the currently selected preset for each category. We briefly summarized the metadata described in each preset category as Electronic Supplementary Material Table S2

In this work, we also implemented functionality in GlycoPOST to register metadata about liquid chromatography experiments according to the MIRAGE proposed guidelines. UniCarb-DR had originally included such functionality which was implemented before the MIRAGE LC guidelines were developed. Therefore, this functionality was added to GlycoPOST to prevent the loss of the existing metadata in UniCarb-DR that describes high-performance liquid chromatography (HPLC) experiments. GlycoPOST manages metadata complementing experimental data by dividing them into categories called “presets,” which are filled out and stored in advance of raw file upload. When submitting experimental data later, researchers can select a preset for each category, saving them the hassle of repeatedly entering descriptions of experiments performed under the same conditions. Therefore, we have introduced a new preset category in GlycoPOST that allows users to describe liquid chromatography experimental information according to MIRAGE liquid chromatography guidelines. Figure 3a shows a screenshot of the web form that allows users to fill in and register presets for the category, allowing researchers to register this type of experimental information in a web browser. Furthermore, GlycoPOST has a feature that allows users to fill in metadata into an Excel spreadsheet file and upload it to be imported as presets for each category in GlycoPOST. We have also extended this functionality to allow metadata about liquid chromatography experiments to be entered through a spreadsheet file instead of a web form, and to extract and register that data as a preset in GlycoPOST.

Figure 3b illustrates a screenshot of the GlycoPOST file upload screen. Users can select presets for each category, such as “Sample preparation” or “Ion sources,” and assign these as metadata to each file they upload. We have updated this form to include a newly introduced category for liquid chromatography so that such metadata can be attached to experimental data. Since these metadata assigned to experimental data can be retrieved by external servers using GlycoPOST's API once the data is published on GlycoPOST, we have also updated the UniCarb-DR system to register this type of metadata.

GlycoPOST currently accepts metadata for experiments that are compliant with the MIRAGE sample preparation, liquid chromatography, and mass spectrometry guidelines. Since these guidelines are only for glycomics, GlycoPOST currently cannot properly accept metadata about other experiments, such as glycoproteomics experiments. However, GlycoPOST has the potential to be extended to accept glycoproteomics experimental data by adding support for various metadata in the same way that it supports metadata for liquid chromatography experimental information. Once GlycoPOST is able to accept such types of experimental data, it will be possible to extract glycopeptide identification results from the experimental data and automatically register them to GlyComb [28], the glycoconjugate data repository, for example. In this way, GlycoPOST is expected to be able to function more comprehensively as a fundamental platform for multi-omics analysis in the field of glycobiology.

### MiniCarb-Viewer: identification result visualization tool during the embargo period

We have also developed a visualization feature similar to the one in UniCarb-DR, called MiniCarb-Viewer, for files in projects under embargo and have incorporated it into GlycoPOST. This allows only specific users access to the data, in addition to the submitter of the experimental data, to visually review the identification results.

**Fig. 4:**
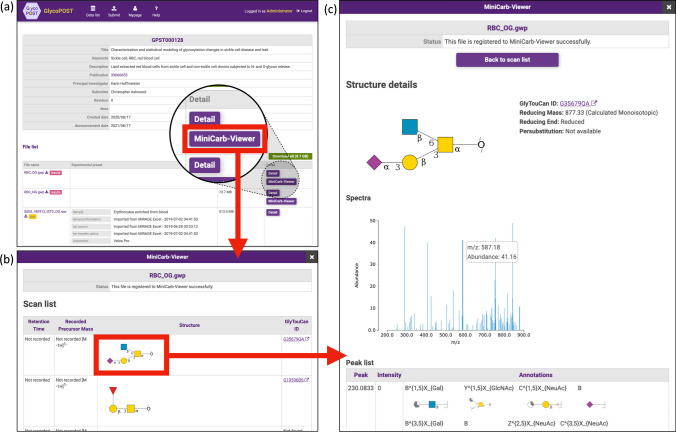
Visualization of identification results by MiniCarb-Viewer in the project preview screen of GlycoPOST. The details of each screen from (**a**) to (**c**) in this figure are described in the main text in the “Results and discussion” section

Figure 4 shows the procedure for visualizing a GlycoWorkbench file on the project preview screen during the peer review period using MiniCarb-Viewer. GlycoPOST can issue a dedicated URL for the preview of the submitted data so that only the reviewers can access the submitter's data during the peer review period. Reviewers can access the URL via a web browser and enter a PIN code provided by the submitter to access the preview screen (Fig. 4a). We have updated this preview screen and placed a “MiniCarb-Viewer” button to launch MiniCarb-Viewer on the corresponding row of the file list if a GlycoWorkbench file is present in the list of files. By clicking this button, a modal window is displayed, which lists a series of identification results, that is, the elution time, precursor ion mass, glycan structure, and if present, the GlyTouCan ID assigned to that structure for each identification result contained in the file in the same way as UniCarb-DR (Fig. 4b). When the reviewer clicks on an image of a glycan structure representing one of the identification results from the list, the detailed information screen corresponding to that identification result is displayed (Fig. 4c). In this screen, the image of the glycan structure representing the identification result is displayed, the spectrum can be visualized with the spectrum viewer, and the possible fragments of the glycan structure annotated for each peak can be displayed.

For submitters, MiniCarb-Viewer can also be used to preview how a GlycoWorkbench file will be displayed in UniCarb-DR before the file is registered into it. If a GlycoWorkbench file is not visualized as expected on MiniCarb-Viewer, it is possible to update the file to be submitted by utilizing the existing GlycoPOST project revision mechanism. After a project has been revised, a preview URL for the revised project can be issued, and the updated identification result file can again be visualized in MiniCarb-Viewer by accessing that URL.

## Conclusions

Unlike proteins, glycans have a branched structure, making it challenging to determine their structure. Moreover, residues at the same position in the same protein sequence may have different glycan structures attached. This heterogeneity makes it extremely hard to determine the glycan molecule. In this context, mass spectrometry has remained an essential tool in glycobiology to gain insight into the structure of glycan molecules in biological samples. Moreover, data repositories that continue to accumulate data obtained by such experimental methods are also crucial as a foundation to enable comprehensive reanalysis of experimental data by computers.

Although GlycoPOST and UniCarb-DR are both data repositories in glycomics with different characteristics, this study has enhanced their interoperability so that submitters of experimental data can submit their data through a single data submission system and automatically cross-reference between corresponding entries in the respective data repositories. As a result, users can now search across both data repositories for raw mass spectrometry data with similar identification results and narrow down the candidate files, which was not possible before.

In the future, glycan-related data from glycomics and glycoproteomics experiments are expected to be increasingly produced as the performance of mass spectrometers improves. It is expected that GlycoPOST and UniCarb-DR can be utilized as indispensable tools to elucidate the effects of glycosylation in organisms as an important foundation to promote informatics-based multi-omics analysis by continuously accumulating these data and applying machine learning and other techniques.

## Supplementary Information

Below is the link to the electronic supplementary material.

Supplementary file1 (DOCX 23 KB)

## Data Availability

The manuscript contains all the data described within the text.
