# Supplementary material for: Cooperation of GlycoPOST and UniCarb-DR towards a comprehensive glycomics data repository workflow
Source: Anal Bioanal Chem. 2024 Nov 29;417(5):1015–23. doi: 10.1007/s00216-024-05673-3 (PMC11782440; doi:10.1007/s00216-024-05673-3)
Supplement: Supplementary file 1 — Supplementary file1 (DOCX 23 KB) [file 216_2024_5673_MOESM1_ESM.docx]

Table S1 A list of 34 GlycoWorkbench files retrieved from GlycoPOST registered with UniCarb-DR as of August 25, 2024

| **GlycoPOST project ID** | **Project title** | **GlycoWorkbench file name** |
| --- | --- | --- |
| GPST000009 | Atlantic Salmon mucus from skin, pyloric caeca and distal intestine | Tasmanian MS2 list.gwp |
| GPST000024 | Discrimination of Isomers of Released N- and O-Glycans Using Diagnostic Product Ions in Negative Ion PGC-LC-ESI-MS/MS | Annotated_spectra.gwp |
| GPST000029 | Standardization of PGC-LC-MS-based glycomics for sample specific glycotyping | Annotated_OG_structures.gwp |
|  |  | Annotated_NG_Structures_update.gwp |
| GPST000030 | Reference glycan structure libraries of primary human cardiomyocytes and pluripotent stem cell-derived cardiomyocytes reveal cell-type and culture stage-specific glycan phenotypes | hiPSC_CM_OG.gwp |
|  |  | hiPSC_CM_NG.gwp |
|  |  | hiPSC_CM_CultureComponents.gwp |
|  |  | primary_CMRV_OG.gwp |
|  |  | primary_CMRV_NG.gwp |
| GPST000038 | UniCarb-DR | GlyoWorkbench file .gwp |
| GPST000052 | Sequencing of heparan sulfate | Structures #1 to #36 and HS1 and HS2.gwp |
| GPST000060 | Core-2 O-glycans are required for galectin-3 interaction with synovial lubricin | bPRG4_Tina.gwp |
| GPST000128 | Characterization and statistical modeling of glycosylation changes in sickle cell disease and trait | RBC_NG.gwp |
|  |  | RBC_OG.gwp |
| GPST000171 | N-glycans from Mayaro virus | Nglycans_from_MAYV_UnicarbDR.gwp |
| GPST000182 | Cardicola forsteri glycome | Supplementary_File_3.gwp |
| GPST000183 | Altered glycoproteome of MLL-rearranged B-cell precursor acute lymphoblastic leukemia | Ngly_annotatedspectra_MLLpatients.gwp |
|  |  | GWB Ogly LA patients.gwp |
| GPST000185 | Glycome of Human Nigrostriatal Tissue and its Alteration in Parkinson's Disease | 210525_brain_mirage.gwp |
| GPST000192 | Sulfation of O-glycans on mucin-type proteins from serous ovarian epithelial tumors | 210528ovarian_final.gwp |
| GPST000198 | Sulfation of O-glycans on mucin-type proteins from serous ovarian epithelial tumors | 210602Gal3ST4core2.gwp |
|  |  | 210602Gal3ST2core2.gwp |
|  |  | 210602CHST1core2.gwp |
| GPST000199 | Sulfation of O-glycans on mucin-type proteins from serous ovarian epithelial tumors | 210607GAL3ST4.gwp |
|  |  | 210607CHST1.gwp |
| GPST000211 | O-glycans released from platelet releasate samples activated with thrombin (0.2 U/mL) | ML_structures+linkages.gwp |
| GPST000279 | Head and Neck Cancer N-glycome traits are cell line and HPV-status dependent | Annotation_Complex.gwp |
|  |  | Annotation_Oligomannose.gwp |
| GPST000307 | The intestinal O-glycome is modified by reactive oxygen species deficiency | Saldova_Mouse_ileum.gwp |
|  |  | Saldova_Mouse_colon.gwp |
| GPST000316 | Sialylation is required for Head and Neck Cancer cell migration | Annotation_O glycan.gwp |
| GPST000334 | O-glycans from Chinese salmon embryonic cell line CHSE-214 | 2023_CHSE214.gwp |
| GPST000335 | O-glycans from rainbow trout gill cell line RTgillW1 | 2023_RTGillW1.gwp |
| GPST000431 | Swift Universal Glycan Acquisition (SUGA) Enables Quantitative Glycan Profiling Across Diverse Sample Types | SUGA_NG.gwp |

Table S2 A list of preset categories (metadata types) that can be associated with mass spectrometry data in GlycoPOST and UniCarb-DR as of October 12, 2024

| **Preset category name** | **Description** |
| --- | --- |
| Sample preparation | The sample preparation preset category is designed to include all aspects of sample generation, purification and modifications of the biological and/or synthetic material analyzed. Users input biologically derived material and/or chemically derived material as sample origin, and enzymatic and/or chemical treatments as sample processing for isolation. In addition, enzymatic and/or chemical modifications, and purification steps are needed to be registered. This preset category follows the MIRAGE sample preparation guidelines. |
| Liquid chromatography | The liquid chromatography preset category includes full information about the liquid chromatography experiment, including the HPLC equipment, the column's properties, pre- and post-processing, and exoglycosidase treatments. This preset category follows the MIRAGE liquid chromatography analysis guidelines. |
| General features | In this preset category, global descriptions in mass spectrometry experiments are included, such as the used instrumentation, any particular customizations, and general instrument control parameters such as instrument control softwares. This also includes the software name and version information. This preset category follows the “general features” section of the MIRAGE mass spectrometric analysis guidelines. |
| Ion sources | The ion sources preset category is used for summarizing all the parameters for ion generation including controls of in-source fragmentation, the degree of fragmentation, as well as other more common parameters such as capillary voltage or laser intensity settings. This preset category follows the “ion sources” section of the MIRAGE mass spectrometric analysis guidelines. |
| Ion transfer optics | The ion transfer optics preset category requires instrumental details related to the processes after ions are generated such as transport, gas phase reactions and detection of ions. This preset category follows the “ion transfer optics” section of the MIRAGE mass spectrometric analysis guidelines. |
| Spectrum and peak list generation and annotation | This preset category is used to describe the software used to generate peak list files from mass spectrometry raw data files and software and/or databases used to annotate each spectrum are needed to be input. This preset category follows the “spectrum and peak list generation and annotation” section of the MIRAGE mass spectrometric analysis guidelines. |
